# (R,R)-BD-AcAc2 Mitigates Chronic Colitis in Rats: A Promising Multi-Pronged Approach Modulating Inflammasome Activity, Autophagy, and Pyroptosis

**DOI:** 10.3390/ph16070953

**Published:** 2023-07-03

**Authors:** Sameh Saber, Mohannad Mohammad S. Alamri, Jaber Alfaifi, Lobna A. Saleh, Sameh Abdel-Ghany, Adel Mohamed Aboregela, Alshaimaa A. Farrag, Abdulrahman H. Almaeen, Masoud I. E. Adam, AbdulElah Al Jarallah AlQahtani, Ali M. S. Eleragi, Mustafa Ahmed Abdel-Reheim, Heba A. Ramadan, Osama A. Mohammed

**Affiliations:** 1 Department of Pharmacology, Faculty of Pharmacy, Delta University for Science and Technology, Gamasa 11152, Egypt; sampharm81@gmail.com; 2 Department of Family Medicine, College of Medicine, University of Bisha, Bisha 61922, Saudi Arabia; malamri@ub.edu.sa; 3 Department of Child Health, College of Medicine, University of Bisha, Bisha 61922, Saudi Arabia; jalfaifi@ub.edu.sa; 4 Department of Clinical Pharmacology, Faculty of Medicine, Ain Shams University, Cairo 11566, Egypt; lobnasaleh_80@yahoo.ca; 5 Department of Clinical Pharmacology, Faculty of Medicine, Mansoura University, Mansoura 35516, Egypt; samghany@mans.edu.eg; 6 Human Anatomy and Embryology Department, Faculty of Medicine, Zagazig University, Zagazig 44519, Egypt; amaboregela@zu.edu.eg; 7 Basic Medical Sciences Department, College of Medicine, University of Bisha, Bisha 61922, Saudi Arabia; 8 Department of Histology and Cell Biology, Faculty of Medicine, Assiut University, Assiut 71515, Egypt; alshaima@aun.edu.eg; 9 Department of Anatomy, College of Medicine, University of Bisha, Bisha 61922, Saudi Arabia; 10 Department of Pathology, College of Medicine, Jouf University, Sakaka 72388, Saudi Arabia; ahalmaeen@ju.edu.sa; 11 Department of Medical Education and Internal Medicine, College of Medicine, University of Bisha, Bisha 61922, Saudi Arabia; mieadam@ub.edu.sa; 12 Department of Internal Medicine, Division of Dermatology, College of Medicine, University of Bisha, Bisha 61922, Saudi Arabia; aaljarallah@ub.edu.sa; 13 Department of Microbiology, College of Medicine, University of Bisha, Bisha 61922, Saudi Arabia; ameleragi@ub.edu.sa; 14 Department of Pharmaceutical Sciences, College of Pharmacy, Shaqra University, Shaqra 11961, Saudi Arabia; 15 Department of Pharmacology and Toxicology, Faculty of Pharmacy, Beni-Suef University, Beni Suef 62521, Egypt; 16 Department of Microbiology and Immunology, Faculty of Pharmacy, Delta University for Science and Technology, Gamasa 11152, Egypt; hebaaa.aadel@gmail.com; 17 Department of Clinical Pharmacology, College of Medicine, University of Bisha, Bisha 61922, Saudi Arabia

**Keywords:** ulcerative colitis, ketone esters, (R,R)-BD-AcAc2, NLRP3 inflammasome, pyroptosis, autophagy, gut dysbiosis

## Abstract

Ulcerative colitis is a chronic and incurable form of inflammatory bowel disease that can increase the risk of colitis-associated cancer and mortality. Limited treatment options are available for this condition, and the existing ones often come with non-tolerable adverse effects. This study is the first to examine the potential benefits of consuming (R,R)-BD-AcAc2, a type of ketone ester (KE), and intermittent fasting in treating chronic colitis induced by dextran sodium sulfate (DSS) in rats. We selected both protocols to enhance the levels of β-hydroxybutyrate, mimicking a state of nutritional ketosis and early ketosis, respectively. Our findings revealed that only the former protocol, consuming the KE, improved disease activity and the macroscopic and microscopic features of the colon while reducing inflammation scores. Additionally, the KE counteracted the DSS-induced decrease in the percentage of weight change, reduced the colonic weight-to-length ratio, and increased the survival rate of DSS-insulted rats. KE also showed potential antioxidant activities and improved the gut microbiome composition. Moreover, consuming KE increased the levels of tight junction proteins that protect against leaky gut and exhibited anti-inflammatory properties by reducing proinflammatory cytokine production. These effects were attributed to inhibiting NFκB and NLRP3 inflammasome activation and restraining pyroptosis and apoptosis while enhancing autophagy as revealed by reduced p62 and increased BECN1. Furthermore, the KE may have a positive impact on maintaining a healthy microbiome. To conclude, the potential clinical implications of our findings are promising, as (R,R)-BD-AcAc2 has a greater safety profile and can be easily translated to human subjects.

## 1. Introduction

Ulcerative colitis is a chronic inflammatory bowel disease (IBD) that affects a large number of people; unfortunately, it is not curable at present. According to an investigation into the worldwide impact of IBDs, there was a significant 47.45% increase in the estimated number of cases between 1990 and 2019 [1]. The total number of individuals living with IBDs globally is estimated to exceed 6.8 million [2]. The reported estimates indicate that the risk of colorectal cancer (CRC) in patients with ulcerative colitis (UC) is approximately 2% after 10 years, 8% after 20 years, and 18% after 30 years of having the disease [3]. According to a comprehensive cohort study conducted in Denmark, individuals with severe microscopic colitis were found to have a higher risk of mortality and colorectal cancer [4].

In addition to reducing the quality of life, patients with this condition are at a higher risk of colon cancer. While there are treatments available for this debilitating disease, they only provide temporary relief and patients are likely to experience relapses over time. Moreover, these treatments can have adverse effects and are often insufficient in achieving long-term remission without maintenance therapy. For many patients, surgery is also a reality, underscoring the urgent need for more effective treatments [5]. Risk factors for ulcerative colitis include genetic susceptibility and factors that influence the gut microbiota, such as antibiotic use and dietary changes, for example, extensive consumption of processed foods [6].

The abnormal activation of the nucleotide-binding oligomerization domain-like receptor protein 3 (NLRP3) inflammasome is a major contributor to the development of chronic colitis, and therapeutic interventions targeting NLRP3 have demonstrated significant efficacy in delaying or preventing disease onset [7]. Inflammasomes are a class of cytosolic protein complexes that can detect a variety of stressors, exogenous pathogens, and endogenous danger signals, triggering activation of caspase-1, and subsequent production of IL-1β and IL-18, thereby initiating the inflammatory process. The priming signal required for inflammasome activation converges on the activation of nuclear transcription factor kappa B (NFκB) and the transcriptional induction of NLRP3 and pro-IL-1β. The activating signal, which may be a danger signal, can directly activate inflammasome assembly [8]. Several mechanisms have been proposed to explain the activation of the NLRP3 inflammasome, including the generation of reactive oxygen species (ROS) [9]. Moreover, downstream of the NLRP3 inflammasome activation, gasdermin D (GSDMD) cleavage and membrane pore-formation has also been observed [10]. The process of pyroptosis, a highly inflammatory type of programmed cell death, is largely dependent on the cleavage of GSDMD, as this cleavage releases the gasdermin D N-terminal fragment (NGSDMD) which carries inherent pyroptosis-inducing capabilities [11]. In addition, The NLRP3 inflammasome has been identified as playing a significant role in coordinating host physiology and immunity, and researchers are actively studying its interactions with the gut microbiota [12]. Recent studies have suggested that dysregulation of the NLRP3 inflammasome in response to changes in the gut microbiota could contribute to the development of chronic colitis [13].

Autophagy is a vital cellular process that removes unnecessary or dysfunctional components through a regulated lysosome-dependent mechanism [14]. It is involved in several pathological processes and plays a significant role in various diseases [15]. Recent research indicates that autophagy dysregulation in intestinal epithelial cells (IECs) contributes to the development and progression of IBDs [16]. Additionally, autophagy has emerged as a potential drug target for managing colitis [17,18] and colitis-associated cancer [19,20]. In particular, targeting autophagy ablation in IECs increases epithelial apoptosis and aggravates inflammatory pathology [21]. These findings imply that manipulating autophagy may be a viable therapeutic approach for the management of chronic colitis.

β-hydroxybutyrate is a ketone body metabolite primarily produced by the brain, liver, heart, and skeletal muscles during caloric restriction, fasting, or a low-carbohydrate ketogenic diet (KD) [22]. Additionally, β-hydroxybutyrate and its producing stimuli, such as fasting and KD, have been shown to possess the ability to improve pro-inflammatory responses. Studies have demonstrated that β-hydroxybutyrate can alleviate pro-inflammatory responses in Parkinson’s disease both in vitro and in vivo [23]. Several studies have shown the beneficial effects of intermittent fasting and calorie restriction on mitigating neuroinflammation and sickness behavior induced by lipopolysaccharide [24,25]. Moreover, a low-carbohydrate KD has been found to reduce tissue inflammatory responses in both juvenile and adult rats [26]. These effects are attributed to the ability of β-hydroxybutyrate to inhibit the NLRP3 inflammasome, which reduces the production of pro-inflammatory cytokines [22].

The brief half-life of β-hydroxybutyrate brings up concerns about its practicality as a potential treatment for humans. Moreover, the therapeutic use of this approach in a clinical environment may not be financially feasible. There have been cases where β-hydroxybutyrate was administered orally to two infants aged six months with persistent hyperinsulinemic hypoglycemia for 5 and 7 months. Despite receiving high doses of up to 32 g/day, these patients reportedly tolerated the treatment well without experiencing any adverse effects [27].

However, ketone esters (KEs), such as (R,R)-BD-AcAc2, offer an alternative source of ketones to boost blood β-hydroxybutyrate levels. Once ingested, KEs are broken down and metabolized, releasing ketone bodies into the bloodstream. This mimics the effects of fasting or adhering to a KD by increasing plasma β-hydroxybutyrate levels. Notably, KE supplementation can significantly enhance plasma β-hydroxybutyrate levels, even when consuming a regular diet. The effects of KEs appear to depend on the dosage and can endure for several hours after ingestion. Researchers are exploring the potential therapeutic benefits of KEs in conditions like epilepsy [28] and Alzheimer’s disease [29], where increasing ketone levels could be advantageous. Some individuals also use KEs to achieve nutritional ketosis without following a strict ketogenic diet. Thus, consuming KEs can be an effective pharmacological approach to raising plasma β-hydroxybutyrate levels.

In addition, It has been suggested that β-hydroxybutyrate may regulate the gut microflora through indirect mechanisms that have yet to be fully understood [30]. While it is widely believed that β-hydroxybutyrate plays a crucial role in the gut microbiome, research directly studying its effects on the gut microflora is still very limited and its mechanism is not well characterized. However, studies have shown that KD can impact gut microbiota composition [31]. Therefore, it is possible that both intermittent fasting and KE consumption, as methods to boost β-hydroxybutyrate production, may lead to β-hydroxybutyrate-induced changes in the microbiome composition. Interestingly, the present study may help to shed light on the potential implications of these changes.

To avoid the need for dietary changes, supplements like KEs have been developed to mimic the effects of ketosis, as an alternative to KD. However, the use of exogenous ketone supplements for treating ulcerative colitis in humans and animals has not been thoroughly and directly evaluated in trials. Although the current evidence indicates that KDs can be a feasible approach, further research is urgently needed to determine the optimal methods, understand the underlying mechanisms, establish dosage and safety guidelines, and promote the widespread adoption of this strategy for treating colitis.

In this study, we have introduced a novel approach and potential treatment option that could improve the chances of long-lasting success against a prevalent type of IBD. Our research has presented preclinical data and explained the molecular mechanism behind utilizing the KE known as (R,R)-BD-AcAc2, for the development of a new IBD therapy. This treatment approach involves targeting the NLRP3 inflammasome and its downstream signaling molecules, inhibiting pyroptosis and inducing autophagy. We conducted preliminary trials to determine doses of (R,R)-BD-AcAc2 that successfully elevate blood levels of β-hydroxybutyrate to mimic a state of nutritional ketosis. Furthermore, 16 h of intermittent fasting in rats succeeded in boosting blood levels of β-hydroxybutyrate, mimicking a state of early ketosis.

This study is the first to examine the potential advantages of consuming (R,R)-BD-AcAc2, a type of KE, in comparison to intermittent fasting for treating chronic colitis induced by dextran sodium sulfate (DSS) in rats. Both protocols were selected to enhance the levels of β-hydroxybutyrate. Our findings provide important insights into a promising multi-pronged approach for achieving long-lasting remission in chronic colitis. The potential clinical implications of our findings are bolstered by the greater safety profile of KEs, which enhances their translatability to human subjects.

## 2. Results

### 2.1. The Impact of KE Ingestion and Intermittent Fasting on the Microscopic Characteristics of Chronic Colitis Induced by DSS

**Figure 1:**
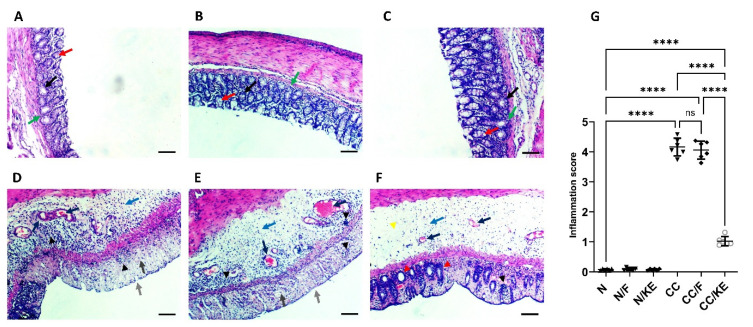
The impact of KE ingestion and intermittent fasting on the microscopic characteristics of chronic colitis induced by DSS. A histopathological evaluation was conducted on the N, N/F, and N/KE groups (**A**–**C**, respectively), which demonstrate normal colonic gland structure characterized by rounded mucus-secreting glands (black arrows), with goblet cells present (red arrows). Additionally, normal mucosal architecture is observed with normal crypt bases and arrangement (green arrows). However, CC (**D**) and CC/F (**E**) groups exhibit submucosal edema (blue arrow), inflamed mucosa (light gray arrow), distorted architecture, great destruction of colonic glands, and loss of goblet cells (dark gray arrow). Submucosal congestion and deposition of fibrotic tissue are evident (dark blue arrows). Furthermore, there are focal and diffuse inflammatory infiltrates of lymphocytes, macrophages, eosinophils, and plasma cells in the mucosal and submucosal layers (black arrowheads). Panel (**F**) indicates an improvement in the structure of the mucosa in the ulcerated area of the CC/KE group. The appearance of glands and crypts (red arrowheads) indicates improved mucosal structure, with a lower level of focal inflammatory cell infiltration (black arrowhead) and clear submucosa with a very low degree of inflammatory cell infiltration (yellow arrowhead), although edema persisted (blue arrow). Additionally, there are less congestion and collagen deposition in the submucosa (black arrows). The scale bar is 100 µm. The inflammation score is shown in panel (**G**), with significance levels indicated by pairwise comparisons. ****, *p* < 0.0001; ns, non-significant.

As depicted in Figure 1, the histopathological assessment of the N, N/F, and N/KE groups (A, B, and C) revealed regular rounded mucus-secreting colonic glands with goblet cells, indicating normal mucosal architecture with normal crypt bases and arrangement. In contrast, sections from the CC and CC/F groups (Dand E) displayed submucosal edema, inflamed mucosa, distortion of architecture, complete destruction of colonic glands, and loss of goblet cells. Submucosal congestion and fibrotic tissue deposition were also observed, along with mucosal and submucosal focal and diffuse inflammatory infiltrates of lymphocytes, macrophages, eosinophils, and plasma cells. The ulcerated area from the CC/KE group (F) showed improvement in the structure of the mucosa, with the appearance of glands and crypts, a lower level of focal inflammatory cell infiltrate, and a clear submucosa of very low degree of inflammatory cell infiltration, while edema still existed. Less congestion and collagen deposition in the submucosa were also observed in this group. These findings were also confirmed upon the assessment of the inflammation score (G).

### 2.2. The Impact of KE Ingestion and Intermittent Fasting on the Mean Percentage Body Weight Change and Colon Weight/Length Ratio

**Figure 2:**
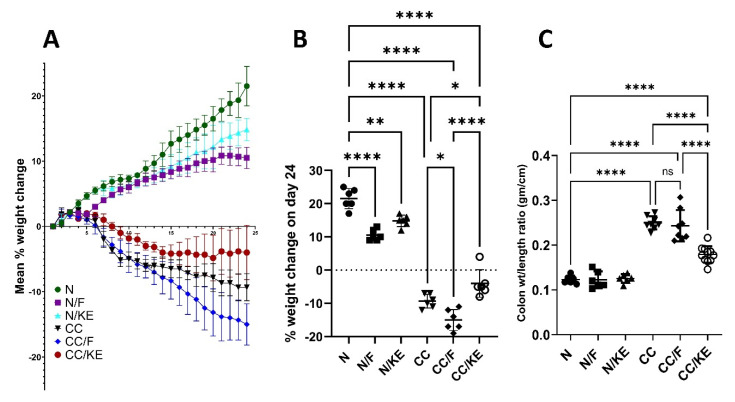
The impact of KE ingestion and intermittent fasting on three parameters: (**A**) the mean percentage body weight change, (**B**) the mean percentage body weight change on day 24, and (**C**) the colon weight/length ratio in rats with chronic colitis induced by DSS. Pairwise comparisons indicate significance levels between groups. *, *p* < 0.05; **, *p* < 0.01; ****, *p* < 0.0001; ns, non-significant.

The percentage change in body weight in animal models can be a useful measure for determining the severity of colitis. This is due to the fact that colitis can promote weight loss through a number of processes, including decreased food intake, malabsorption, and increased energy expenditure as a result of inflammation. We can quantify the activity and severity of a disease objectively by tracking changes in body weight over time. Figure 2A illustrates the progressive body weight changes among different groups during the experimental period, while Figure 2B shows that both fasting and KE ingestion resulted in a significant decrease in body weight compared with normal rats. The study found that fasting resulted in a significant decrease in the percentage of body weight change in rats with chronic colitis when compared with the CC group. Similarly, the ingestion of KE, and not intermittent fasting, led to a significant reduction in the percentage of body weight change in rats with chronic colitis when compared with the CC group. These results suggest that KE ingestion may be effective in preventing body weight loss induced by chronic DSS administration. Additionally, the weight-to-length ratio of the colon is commonly used as an indicator of colitis severity in animal models, particularly in pre-clinical trials of new drugs for ulcerative colitis. Lower final ratios are associated with greater effectiveness in reducing severity. The results of the study demonstrated that KE consumption significantly decreased the final colon weight-to-length ratio in contrast to the CC group of rats, demonstrating KE’s efficacy in lowering the severity of colitis (as shown in Figure 2C).

### 2.3. The Impact of KE Ingestion and Intermittent Fasting on the Disease Activity Index and Macroscopic Damage Index

**Figure 3:**
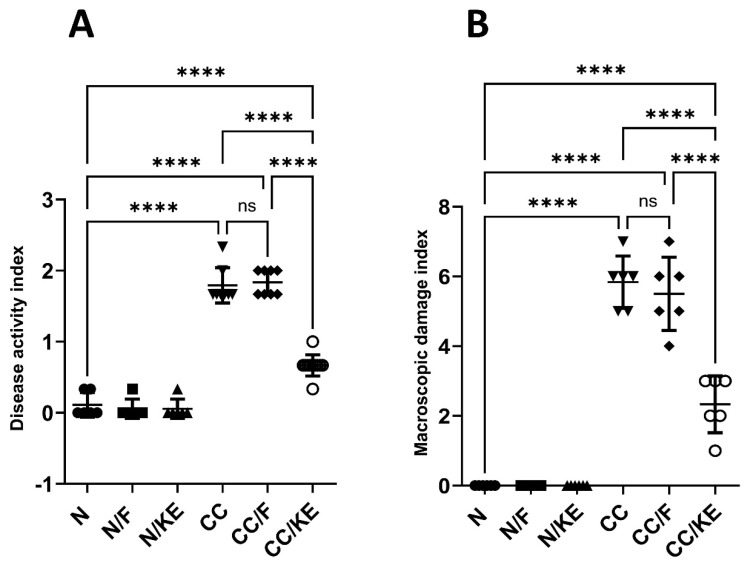
The impact of KE ingestion and intermittent fasting on the disease activity index (**A**) and macroscopic damage index (**B**) in rats with chronic colitis induced by DSS. Pairwise comparisons show the significance levels between the groups. ****, *p* < 0.0001; ns, non-significant.

The DAI provides a composite score of colitis severity based on the selected parameters of weight loss, stool consistency, and rectal bleeding. The DAI supplements other metrics like colon weight-to-length ratio and provides an aggregate quantitative measure of colitis severity from different perspectives to complement other evaluations. Based on our findings as shown in Figure 3A, we observed that the DAI significantly increased in the CC group of rats compared with the normal values. However, the rats from the CC/KE group displayed a significant reduction in the score compared with the CC group. On the other hand, the CC/F group did not show a significant change in the DAI compared with that of the CC group. These results indicate that the KE-induced increases in the β-hydroxybutyrate are significantly linked to a reduction in the colitis severity. This suggests that boosting ketone production helps reduce colitis severity as measured by the DAI.

In addition, the macroscopic damage index provides an additional objective measure of colonic damage that supplements other metrics. It evaluates colitis severity from the perspective of macroscopic colon abnormalities. Our findings (Figure 3B) showed that the MDI in the CC group of rats significantly increased compared with the normal values. When compared with the CC group, however, the rats from the CC/KE group showed a significant decline in scores. The MDI did not significantly vary between the CC group and the CC/F group, on the other hand. These findings suggest that a decrease in the severity of colitis is highly correlated with KE-induced elevations in the β-hydroxybutyrate.

### 2.4. The Impact of KE Ingestion and Intermittent Fasting on the Survival Property

**Figure 4:**
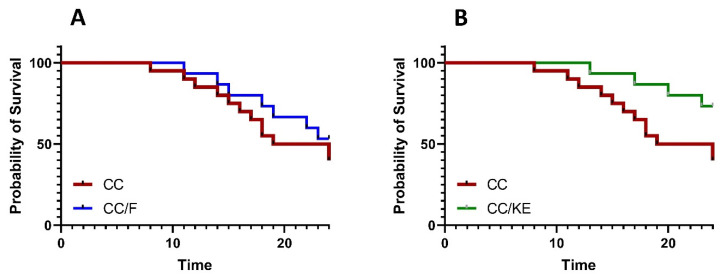
Survival analysis comparisons between the CC group and the CC/F group (**A**) and the CC group and CC/KE group (**B**), show the impact of KE ingestion and intermittent fasting on the survival property in rats with chronic colitis induced by DSS. The log-rank (Mantel–Cox) test was used to determine individual survival analyses. Pairwise comparisons indicate significance levels between groups.

Survival analysis is a significant statistical method for assessing the effectiveness of treatments for colitis. This approach analyzes the time it takes for an event to occur, and a longer time for the event with the experimental treatment indicates a higher level of effectiveness. Additionally, hazard ratios derived from survival analysis measure the risk of experiencing an adverse event, such as death, in one treatment group compared with another. The log-rank (Mantel–Cox) test was used to compare individual survival analyses between the DSS-exposed rats (CC group) and the CC/F or CC/KE groups of rats. The outcomes showed no statistically significant difference in the likelihood of survival between the CC group and the CC/F group (Figure 4A). Contrarily, our research showed that the DSS-exposed rats’ survival rate considerably increased after receiving KE (Figure 4B; *p* = 0.045). Additionally, the CC and CC/KE groups’ hazard ratio (log-rank) was 2.92 (95 percent CI of ratio 1.096 to 7.785). The increased survivability in colitic rats of KE-induced β-hydroxybutyrate plasma levels suggests that increasing ketone availability may be a viable treatment for colitis. The findings suggest that ketones may have anti-inflammatory, bioenergetic, and maybe protective cellular effects that could delay disease progression, lessen the severity, and prolong remission.

### 2.5. The Impact of KE Ingestion and Intermittent Fasting on Oxidative Stress Parameters

**Figure 5:**
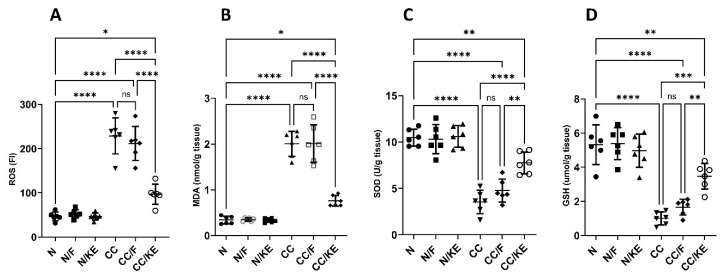
Impact of KE ingestion and intermittent fasting on oxidative stress markers: reactive oxygen species (ROS) (**A**), malondialdehyde (MDA) (**B**), superoxide dismutase (SOD) (**C**), and reduced glutathione (GSH) (**D**) in rats with chronic colitis induced by DSS. Pairwise comparisons indicate significance levels between groups. *, *p* < 0.05; **, *p* < 0.01; ***, *p* < 0.001; ****, *p* < 0.0001; ns, non-significant.

Oxidative stress indicators can offer important insights into the severity and development of colitis. Lipids, proteins, DNA, and other molecules become oxidatively damaged when oxidants and antioxidants are out of equilibrium. Colitis severity is correlated with the degree of oxidative stress. Additionally, in colitis, the levels of lipid peroxidation products such as MDA are elevated. This increase in MDA is indicative of greater oxidative damage to the membranes, which suggests a more severe inflammatory response and compromise to the gastrointestinal barrier. Our results revealed a significant increase in the levels of ROS (Figure 5A) and MDA (Figure 5B) in response to DSS exposure compared with those of the normal rats. In contrast, KE ingestion significantly suppressed the DSS-induced increase in these levels. Enzymatic antioxidants, such as superoxide dismutase (SOD) play a critical role in regulating oxidant levels. Reduced activity of these enzymes impairs their ability to control reactive oxygen/nitrogen species levels, thereby worsening the severity of colitis. Also, during colitis, there is a depletion of the essential antioxidant glutathione (GSH). This results in increased susceptibility of cells to oxidative damage. We revealed a significant reduction in the levels of SOD (Figure 5C) and GSH (Figure 5D) in response to DSS exposure compared with those of the normal rats. In contrast, KE ingestion significantly suppressed the DSS-induced decrease in these levels.

### 2.6. The Impact of KE Ingestion and Intermittent Fasting on the Plasma Levels of Β-Hydroxybutyrate

**Figure 6:**
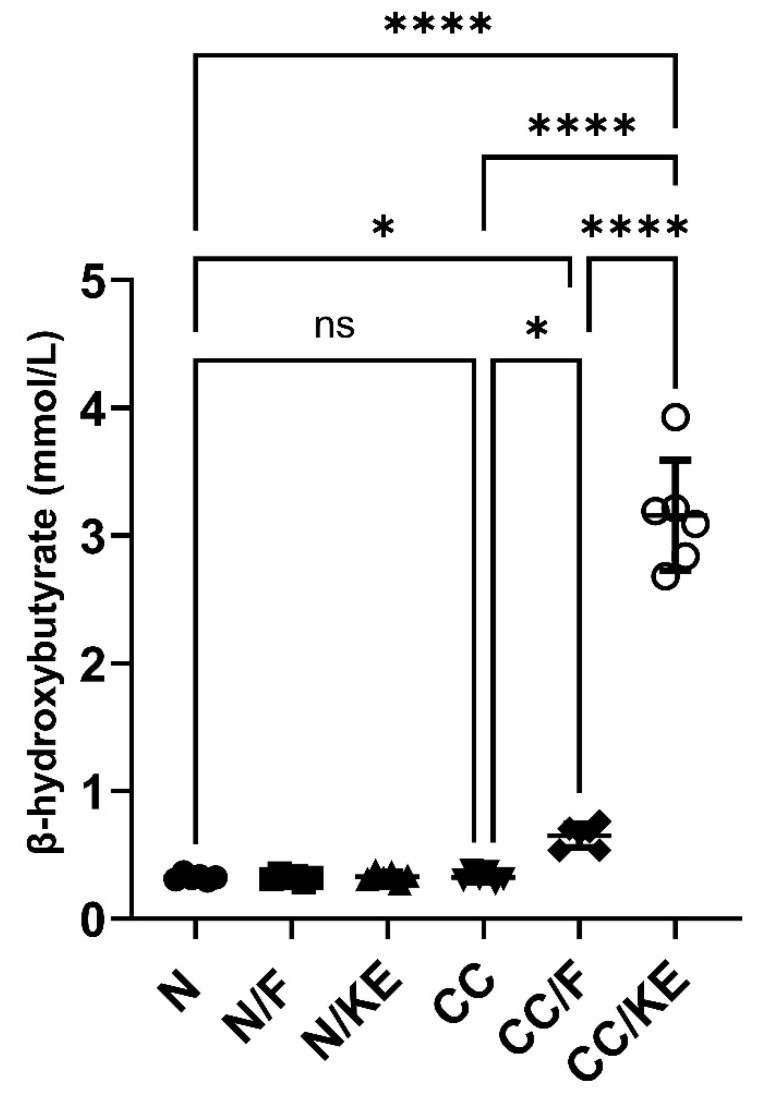
The impact of KE ingestion and intermittent fasting on plasma levels of β-hydroxybutyrate in rats with chronic colitis induced by DSS. Significance levels are indicated by pairwise comparisons. *, *p* < 0.05; ****, *p* < 0.0001; ns, non-significant.

At the end of the fasting period, the selected intermittent fasting protocol resulted in a significant increase in the β-hydroxybutyrate levels compared with normal non-fasting rats (adjusted *p* value = 0.043, 95% CI of difference −0.9895 to −0.01063), indicating a state resembling early ketosis. Conversely, ingestion of KE resulted in a significant increase in the β-hydroxybutyrate levels compared with normal non-fasting rats (adjusted *p* value < 0.0001, 95% CI of difference −3.525 to −2.546) indicating a state resembling nutritional ketosis. In contrast, chronic administration of DSS in the CC rat group did not significantly alter β-hydroxybutyrate levels compared with normal rats. Additionally, intermittent fasting in the CC/F rat group resulted in a significant increase in the levels of β-hydroxybutyrate compared with those of CC rats (adjusted *p* value = 0.02). Similarly, KE ingestion in CC/KE rat group resulted in a significant increase in the levels of β-hydroxybutyrate when compared with those of CC rats (adjusted *p* value < 0.0001). Overall, these findings (illustrated in Figure 6) suggest that both intermittent fasting for 16 h per day and KE ingestion elevated β-hydroxybutyrate levels beyond normal levels, inducing a state resembling early and nutritional ketosis, respectively.

### 2.7. The Impact of KE Ingestion and Intermittent Fasting on Different Cytokines as Inflammation Markers

**Figure 7:**
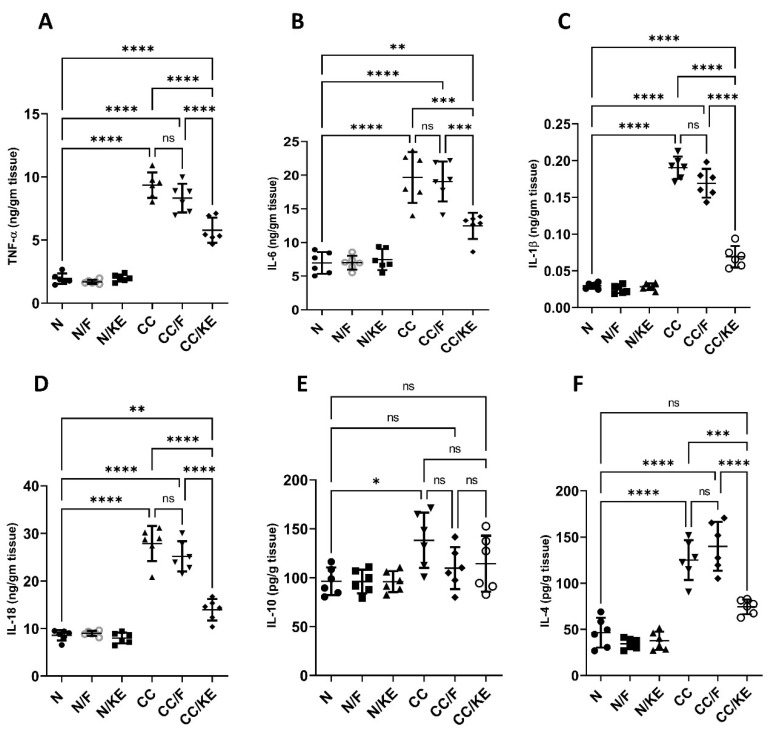
The impact of KE ingestion and intermittent fasting on tumor necrosis factor-alpha (TNF-α) (**A**), IL-6 (**B**), IL-1β (**C**), IL-18 (**D**), IL-10 (**E**), and IL-4 (**F**) levels in rats with chronic colitis induced by DSS. Significance levels are indicated by pairwise comparisons. *, *p* < 0.05; **, *p* < 0.01; ***, *p* < 0.001; ****, *p* < 0.0001; ns, non-significant.

In the CC group, chronic administration of DSS resulted in a significant increase in the levels of pro-inflammatory cytokines TNF-α (Figure 7A), IL-6 (Figure 7B), IL-1β (Figure 7C), and IL-18 (Figure 7D), and a significant increase in the levels of anti-inflammatory cytokines IL-10 (Figure 7E) and IL-4 (Figure 7F), compared with those in normal rats. However, the ingestion of KE had a significant impact on the reversal of DSS-induced changes in cytokine levels in the CC/KE groups, compared with the CC groups. Specifically, TNF-α, IL-6, IL-1β, IL-18, and IL-4 levels were significantly reduced, while IL-10 levels remained unchanged. Chronic colitis is known to be associated with an increase in pro-inflammatory cytokines, which contribute to persistent inflammation and colon tissue damage. The elevation of IL-1β and IL-18 levels suggests inflammasome activation, which is believed to be involved in chronic colitis. The higher levels of IL-10 and IL-4 in CC groups indicated a shift towards a Th2-mediated immune response. These cytokines are produced by various immune cells, including Th2 cells, Tregs, and macrophages, which are activated in chronic colitis. It is worth noting that cytokine levels were not significantly altered in rats that underwent intermittent fasting and were treated with DSS, compared with non-fasting rats that received DSS treatment.

### 2.8. The Impact of KE Ingestion and Intermittent Fasting on the Activities of Each Mpo, Nfκb Dna Binding, Caspase-1, and the Levels of Active Caspase-3

**Figure 8:**
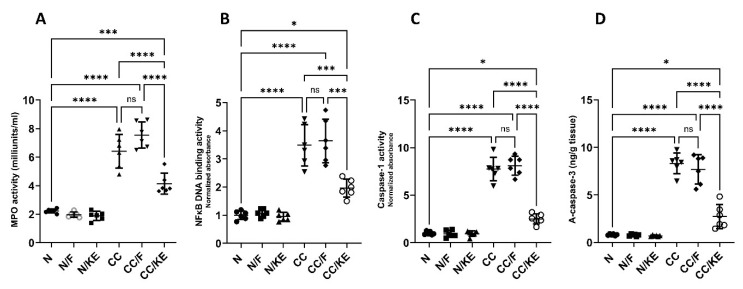
The impact of KE ingestion and intermittent fasting on the activities of each myeloperoxidase (MPO) (**A**), nuclear transcription factor kappa B (NFκB) DNA binding (**B**), caspase-1 (**C**), and the levels of active caspase-3 (**D**) in rats with chronic colitis induced by DSS. Significance levels are indicated by pairwise comparisons. *, *p* < 0.05; ***, *p* < 0.001; ****, *p* < 0.0001; ns, non-significant.

In chronic colitis, sustained activation of neutrophils leads to the release of MPO, which contributes to tissue damage by generating ROS and other reactive intermediates. The increased levels of NFκB in chronic colitis promote the sustained production of pro-inflammatory cytokines, thereby perpetuating the inflammatory response. NFκB activates the expression of genes involved in cytokine production, such as TNF-α, IL-6, and IL-1β, which recruit immune cells and further damage the tissue. In chronic colitis, increased activity of caspase-1 leads to the production of pro-inflammatory cytokines and the perpetuation of the inflammatory response. Caspase-1 processes the bioactivation of IL-1β and IL-18, which stimulate immune cells and promote tissue damage. Similarly, increased activity of caspase-3 in chronic colitis contributes to tissue damage and disease progression by inducing apoptosis and subsequent loss of epithelial cells. Apoptosis of intestinal epithelial cells leads to disruption of the intestinal barrier and increased permeability, allowing luminal antigens and bacteria to enter the mucosa and trigger further inflammation. Additionally, caspase-3-mediated apoptosis of immune cells can contribute to the dysregulation of the immune response and perpetuation of inflammation. Our results revealed that the activities of MPO (Figure 8A), NFκB DNA binding (Figure 8B), and caspase-1 (Figure 8C), and the levels of active caspase-3 (Figure 8D) were significantly increased in the chronic colitis rat group compared with normal rats. However, ingestion of KE significantly reversed the DSS-induced alterations in their levels, while intermittent fasting did not significantly alter their levels in the CC/F group compared with the CC group.

### 2.9. The Impact of KE Ingestion and Intermittent Fasting on the mRNA Expression of ASC and NLRP3

**Figure 9:**
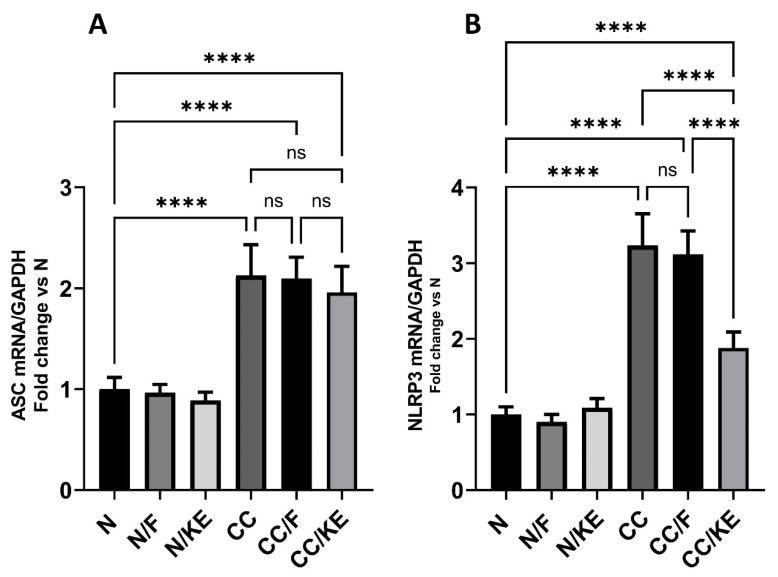
The impact of KE ingestion and intermittent fasting on the levels of the mRNA expression of apoptosis-associated speck-like protein containing a CARD (ASC) (**A**) and nucleotide-binding oligomerization domain-like receptor protein 3 (NLRP3) (**B**) in rats with chronic colitis induced by DSS. Significance levels are indicated by pairwise comparisons. ****, *p* < 0.0001; ns, non-significant.

ASC acts as a linker protein, aiding in the assembly and activation of the inflammasome complex. The protein’s N-terminus contains a caspase recruitment domain (CARD), which recruits and binds caspase-1 to the inflammasome complex, facilitating its activation. This process leads to the cleavage of pro-IL-1β and pro-IL-18 into their active forms. Additionally, ASC comprises a pyrin domain, which interacts with the NLRP3 sensor protein. The activation of the NLRP3 inflammasome occurs in two steps. First, the inflammasome undergoes priming through the activation of NFκB, which results in the upregulation of inflammasome components such as NLRP3. The second step involves the activation of NLRP3 by specific danger signals. This process leads to the assembly of the inflammasome complex and subsequent activation of caspase-1. Chronic colitis can be triggered by various factors that activate the NLRP3 inflammasome. These factors include the release of damage-associated molecular patterns (DAMPs) by damaged epithelial cells and the dysbiosis of the gut microbiome. The activation of the NLRP3 inflammasome results in an increased secretion of IL-1β and IL-18, promoting inflammation, epithelial damage, and the infiltration of immune cells into the colonic mucosa. This exacerbates the inflammation in chronic colitis. Our study found that in rats with chronic colitis, the mRNA expression of ASC (Figure 9A) and NLRP3 (Figure 9B) was significantly higher than in normal rats. However, ingestion of KE significantly reduced the mRNA expression of NLRP3, while ASC mRNA expression remained unchanged in the CC/KE group compared with the CC group. Additionally, there was no significant change in the mRNA expression of ASC and NLRP3 in the CC/F rat group compared with normal rats. These findings suggest that KE may have a therapeutic effect on chronic colitis by modulating the expression of NLRP3, a crucial component of the inflammasome complex.

### 2.10. The Impact of KE Ingestion and Intermittent Fasting on the Levels of NLRP3 and NGSDMD

**Figure 10:**
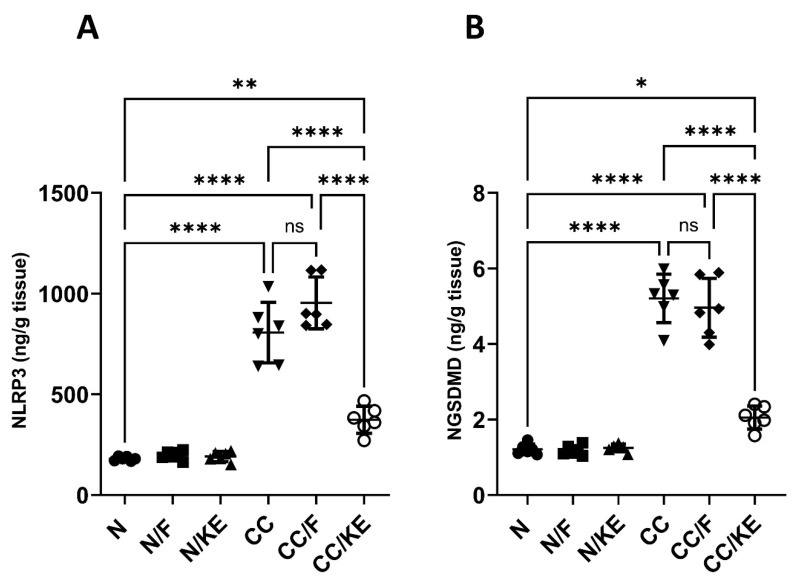
The impact of KE ingestion and intermittent fasting on the levels of nucleotide-binding oligomerization domain-like receptor protein 3 (NLRP3) (**A**) and gasdermin D N-terminal fragment (NGSDMD) (**B**) in rats with chronic colitis induced by DSS. Significance levels are indicated by pairwise comparisons. *, *p* < 0.05; **, *p* < 0.01; ****, *p* < 0.0001; ns, non-significant.

The NLRP3 inflammasome is involved in activating caspase-1, which in turn cleaves GSDMD to generate its active N-terminal fragment known as NGSDMD. This fragment then oligomerizes to form membrane pores leading to cell swelling, membrane rupture, and pyroptotic cell death that allow the release of inflammatory cytokines. Excessive GSDMD cleavage has been linked to heightened pyroptosis, a form of programmed cell death, and inflammation in the colonic mucosa. Our study revealed a significant increase in both NLRP3 (Figure 10A) and NGSDMD (Figure 10B) levels in the CC group compared with the N group. However, the CC/KE group exhibited a significant reduction in these levels due to the consumption of KE compared with the CC group. Notably, intermittent fasting had no significant effect on these levels.

### 2.11. The Impact of KE Ingestion and Intermittent Fasting on the Macroautophagy Markers

**Figure 11:**
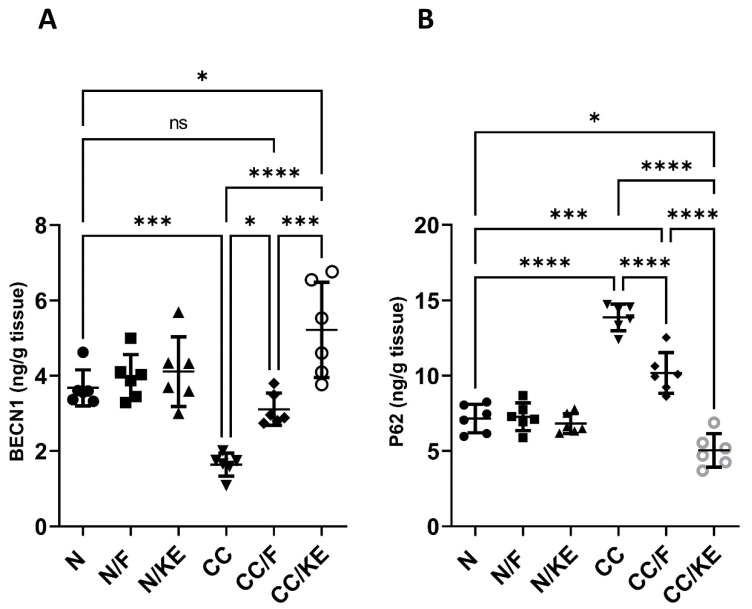
The impact of KE ingestion and intermittent fasting on Beclin-1 (BECN1) (**A**) and sequestosome-1 (p62) (**B**) in rats with chronic colitis induced by DSS. Significance levels are indicated by pairwise comparisons. *, *p* < 0.05; ***, *p* < 0.001; ****, *p* < 0.0001; ns, non-significant.

The activation of macroautophagy in colitis is thought to be an adaptive response to help limit inflammation and tissue injury, indicating it plays a protective role. stimulating macroautophagy may have the potential as a treatment strategy. BECN1 is an important protein involved in the activation of macroautophagy. It forms a protein complex that is required for the initiation of macroautophagy by recruiting other proteins to form the isolation membrane that engulfs cellular components for degradation. BECN1 levels correlate with the activation of macroautophagy. Higher levels of BECN1 generally indicate more induction of the macroautophagy process. On the other hand, P62 is a protein that undergoes selective degradation through macroautophagy. Thus, its levels show an inverse correlation with macroautophagy activation. P62 binds to proteins and organelles that macroautophagy targets for degradation. It interacts with proteins on the autophagosome membrane to facilitate degradation. Once engulfed by the autophagosome, lysosomal enzymes break down p62 and the molecules it binds. Inducing macroautophagy leads to the degradation of more p62 and its targets, resulting in lower p62 levels in the cell. Conversely, impaired macroautophagy cannot effectively degrade p62, leading to its accumulation in the cell. In our study, we detected impaired macroautophagy in the CC rat group compared with normal rats. However, we observed macroautophagy activation in the CC/F group compared with the CC group (BECN1, *p* < 0.02; p62, *p* < 0.0001), as well as in the CC/KE group compared with the CC group (BECN1, *p* < 0.0001; p62, *p* < 0.0001). These findings (as shown in Figure 11A for BECN1 and Figure 11B for p62) suggest that both intermittent fasting and KE consumption induced macroautophagy activation, with KE consumption demonstrating a particularly strong effect.

### 2.12. The Impact of KE Ingestion and Intermittent Fasting on Tight Junction Proteins

**Figure 12:**
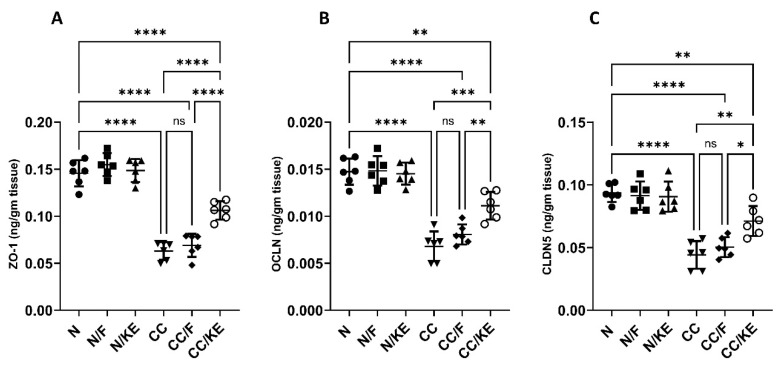
The impact of KE ingestion and intermittent fasting on tight junction proteins zonula occludens-1 (ZO-1) (**A**), occludin (OCLN) (**B**), and claudin-5 (CLDN5) (**C**) in rats with chronic colitis induced by DSS. Significance levels are indicated by pairwise comparisons. *, *p* < 0.05; **, *p* < 0.01; ***, *p* < 0.001; ****, *p* < 0.0001; ns, non-significant.

Maintaining the integrity and barrier function of the intestinal epithelium is a critical function of tight junction proteins. However, chronic colitis can disrupt these proteins, leading to increased intestinal permeability, bacterial translocation, and the release of inflammatory cytokines. In our study, we administered DSS to rats in the CC group, which resulted in a significant decrease in the levels of tight junction proteins ZO-1 (Figure 12A), OCLN (Figure 12B), and CLDN5 (Figure 12C) compared with their baseline levels. Interestingly, KE consumption, rather than intermittent fasting, resulted in significant increases in the levels of these proteins compared with the DSS-treated CC rat group.

### 2.13. Correlation Analysis of the Measured Parameters

**Figure 13:**
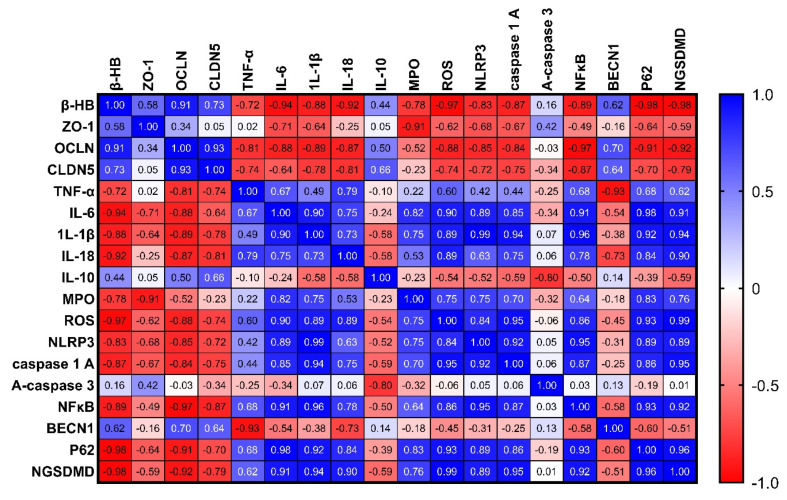
Correlation analysis of the measured parameters in rats with chronic colitis induced by DSS.

As shown in Figure 13, our research findings demonstrate a positive correlation between the levels of β-hydroxybutyrate in rats with chronic colitis and the tight junction proteins OCLN and CLDN5, with a strong tendency towards a positive correlation with ZO-1. This suggests that increasing the levels of β-hydroxybutyrate could be beneficial in reducing gut permeability. In addition, β-hydroxybutyrate may help manage colitis symptoms by stabilizing or upregulating these key barrier proteins. This potential mechanism may explain the beneficial effects of ketogenic diets or ketone supplementation in managing colitis and other forms of intestinal inflammation.

In addition, our study revealed a negative correlation between the levels of β-hydroxybutyrate in rats with chronic colitis and proinflammatory cytokines such as TNF-α, IL-6, IL-1β, and IL-18, with a strong tendency towards a positive correlation with IL-10 levels. This suggests that β-hydroxybutyrate may have anti-inflammatory effects in chronic colitis by reducing the production of proinflammatory cytokines and mitigating the inflammatory response. Additionally, β-hydroxybutyrate may increase the levels of the anti-inflammatory cytokine IL-10 to suppress inflammation and facilitate intestinal healing.

Furthermore, our investigation revealed a negative correlation between the levels of β-hydroxybutyrate in rats with chronic colitis and several inflammatory markers, including MPO activity, ROS production, NLRP3 levels, caspase-1 activity, NFκB DNA binding activity, and the NGSDMD. However, we did not find a significant correlation with active caspase-3 levels. These findings suggest that β-hydroxybutyrate may help mitigate gut inflammation in chronic colitis by inhibiting inflammasome activation and oxidative stress through various mechanisms. Specifically, MPO activity and ROS production were negatively correlated with β-hydroxybutyrate levels, implying that β-hydroxybutyrate may reduce oxidative stress, which is often increased in colitis and contributes to gut inflammation. Moreover, NLRP3 levels, caspase-1 activity, and NFκB DNA binding activity were all negatively correlated with β-hydroxybutyrate levels. These components play a role in the NLRP3 inflammasome activation, which drives intestinal inflammation in colitis by consequent production of IL-1β and IL-18. Thus, the negative correlations suggest that β-hydroxybutyrate may suppress NLRP3 inflammasome activation. In addition, NGSDMD levels were also negatively correlated with β-hydroxybutyrate. NGSDMD release is associated with pyroptosis, a proinflammatory form of programmed cell death. Therefore, lower levels of NGSDMD with higher β-hydroxybutyrate suggest that β-hydroxybutyrate may inhibit pyroptosis. However, our results did not show a significant correlation between caspase-3 activity and β-hydroxybutyrate levels. This indicates that β-hydroxybutyrate’s effects may be specific to inflammasome-related pathways rather than overall apoptosis.

Finally, we revealed a negative correlation between levels of β-hydroxybutyrate and p62, while there is a positive correlation with BECN1. This suggests that β-hydroxybutyrate may have a modulating effect on autophagy in chronic colitis. The negative correlation between β-hydroxybutyrate and p62 indicates that higher levels of β-hydroxybutyrate are associated with lower levels of p62 protein, which is a marker of autophagic flux. Increased levels of p62 typically indicate impaired autophagy, so the lower p62 levels observed with higher β-hydroxybutyrate suggest that β-hydroxybutyrate may enhance autophagy. The positive correlation between β-hydroxybutyrate and BECN1 further supports this idea, as BECN1 is important for initiating autophagy and higher levels of BECN1 promote autophagy. Therefore, the positive correlation between β-hydroxybutyrate and BECN1 suggests that β-hydroxybutyrate may stimulate autophagy by upregulating BECN1. Autophagy helps remove damaged organelles and aggregates to maintain cellular homeostasis, and its impairment has been linked to chronic inflammation, including colitis. By potentially enhancing autophagy through reducing p62 and increasing BECN1, β-hydroxybutyrate may help resolve gut inflammation in colitis. Although the correlations provide insight into potential mechanisms, the observational nature of the correlation means that causation cannot be definitively established.

### 2.14. The Impact of KE Ingestion and Intermittent Fasting on Microbiome Composition

**Figure 14:**
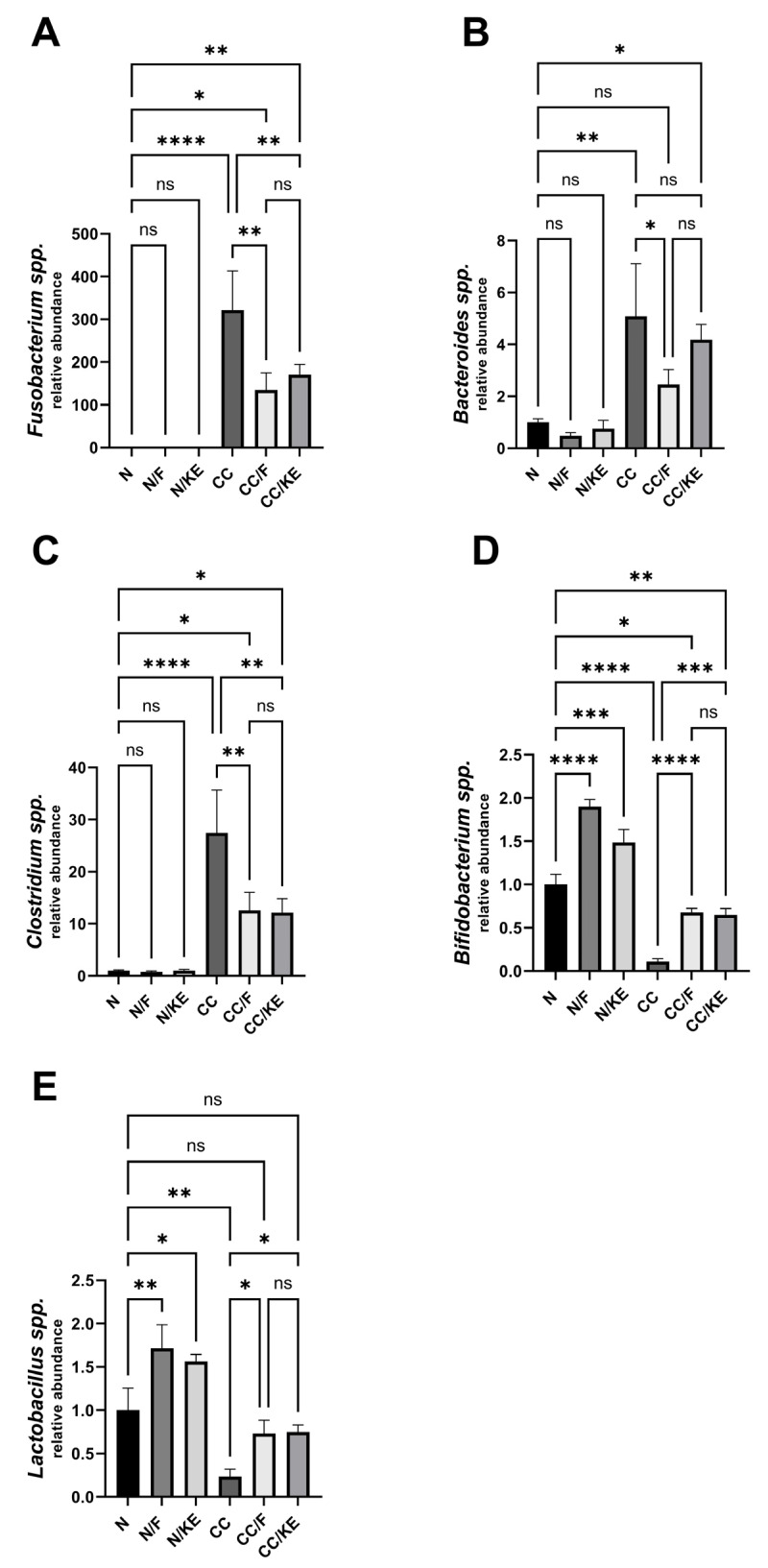
The impact of KE ingestion and intermittent fasting on microbiome composition in rats with chronic colitis induced by DSS: This figure depicts the changes in the microbiome composition of five bacterial species, *Fusobacterium* spp. (**A**), *Bacteroids* spp. (**B**), *Clostridium* spp. (**C**), *Bifidobacterium* spp. (**D**), and *Lactobacillus* spp. (**E**), in response to KE ingestion and intermittent fasting. The significance levels are indicated by pairwise comparisons, and the results suggest significant changes associated with β-hydroxybutyrate boosting on the microbiome composition of these bacterial species. *, *p* < 0.05; **, *p* < 0.01; ***, *p* < 0.001; ****, *p* < 0.0001; ns, non-significant.

Chronic colitis is associated with elevated levels of *Fusobacterium* species, which may contribute to inflammation by invading intestinal cells, activating the NLRP3 inflammasome, and dysregulating the microbiome. Additionally, increased levels of *Bacteroides* have been implicated in some inflammatory bowel diseases such as chronic colitis. The overgrowth of certain *Clostridium* species may also trigger and sustain chronic inflammation in colitis by affecting the intestinal environment and immune system. On the other hand, *Bifidobacteria* are a beneficial bacteria genus that constitutes a significant proportion of the gut microbiota, particularly in the colon. Reduction in *Bifidobacteria* is linked to increased disease severity and poorer outcomes in colitis. Lactobacillus bacteria are generally considered probiotics that may have potential health benefits. However, patients with ulcerative colitis and Crohn’s disease often exhibit lower levels of Lactobacillus than healthy individuals. This observation suggests that lower abundances of *Lactobacillus* may be associated with dysbiosis and chronic inflammation in the gut. In our study, we revealed a significant disruption in the gut composition of the abovementioned bacteria species in the CC rat group. While both intermittent fasting and KE ingestion caused a significant reversal in the relative abundance of these bacteria species compared with the CC group (except for KE in the case of *Bacteroides*), neither regimen could fully restore the normal relative abundance of these bacteria, except for *Lactobacillus,* as depicted in Figure 14A (*Fusobacterium*), B (*Bacteroides*), C (*Clostridium*), D (*Bifidobacteria*), and E (*Lactobacillus*).

## 3. Discussion

Ulcerative colitis is a chronic and incurable form of IBDs that can significantly impair quality of life and increase the risk of colitis-associated cancer and mortality. One major challenge associated with ulcerative colitis is the limited treatment options available, which often come with non-tolerable adverse effects.

In this study, we aimed to shed light on the potential therapeutic value of ketosis/ketone production in managing IBD. Specifically, we investigated the benefits of ketone-based medications or supplements that can increase ketone levels without requiring a restrictive diet. This approach has the potential to improve compliance and safety in the treatment of chronic colitis. To examine this novel treatment method, we investigated the effects of two regimens that increase β-hydroxybutyrate levels: intermittent fasting and the consumption of a specific type of KE called (R,R)-BD-AcAc2. We evaluated their effectiveness in managing chronic colitis induced by DSS in rats.

In accordance with our protocol, rats were administered the KE, resulting in elevated levels of β-hydroxybutyrate, indicating a state resembling nutritional ketosis. Additionally, 16 h of intermittent fasting led to increased levels of β-hydroxybutyrate, suggesting the onset of early ketosis. Interestingly, despite exhibiting modulation of autophagy and correction of dysbiosis, rats subjected to 24 days of intermittent fasting did not show significant coloprotective effects against chronic DSS administration. In contrast, the consumption of KE demonstrated a notable coloprotective property, primarily by modulating the NLRP3 inflammasome and its downstream inflammatory pyroptotic cell death. These results indicate that achieving a state of nutritional ketosis, as opposed to early ketosis, is necessary to confer a coloprotective effect.

It is noteworthy that both protocols demonstrated almost the same impact on the relative abundance of the tested bacteria. This suggests that the correction of dysbiosis is not the primary mechanism through which β-hydroxybutyrate alleviates colitis. Although the intermittent fasting protocol induced only a small increase in autophagy, it seems that this minor change has a negligible effect on colon inflammation. On the other hand, KE consumption was more effective than the intermittent fasting protocol as it exhibited stronger autophagy-inducing capabilities in addition to demonstrating the inactivation of the NLRP3 inflammasome.

In addition to improving disease activity, the consumption of KE resulted in the improvement of colonic macroscopic and microscopic features, as well as a decrease in inflammation scores. Furthermore, KE counteracted the DSS-induced decrease in the percentage of weight change upon experiment completion, reduced the colonic weight-to-length ratio, and increased the survival rate of DSS-insulted rats. Although oxidative stress parameters were not normalized, KE ingestion showed potential antioxidant activities. Along with improving gut microbiome composition, KE consumption improved the levels of tight junction proteins, which protect against leaky gut. Moreover, KE consumption exhibited anti-inflammatory properties by reducing proinflammatory cytokine production.

It is worth noting that our results revealed an increase in the levels of IL-10 and IL-4 in non-treated chronic colitis rats. This result suggests that the body mounts an immunoregulatory response involving IL-10 and IL-4 against chronic colitis, but this response is inadequate to resolve the inflammation. Additionally, this result indicated a shift towards a Th2-mediated immune response, confirming the chronic nature of our model.

The transcriptional regulation of IL-1β and IL-18 is mediated by NFκB, which acts as the priming signal for the activation of the NLRP3 inflammasome. The assembly of the inflammasome complex and subsequent activation of caspase-1 leads to the bioactivation of IL-1β and IL-18, making them key players in the pyroptosis process. The coloprotective effect of KE can be attributed to its ability to inhibit both the priming and activation signals of the NLRP3 inflammasome, thus preventing highly inflammatory pyroptotic cell death. Importantly, KE consumption was found to decrease NFκB DNA binding and downregulate NLRP3, which is also transcriptionally regulated by NFκB. This results in a reduction in caspase-1 activity, ultimately leading to the inhibition of IL-1β and IL-18 bioactivation. This was further confirmed by a reduction in the gasdermin D N-terminal fragment, which is indicative of curbed pyroptosis. During pyroptosis, inflammatory cytokines such as IL-1β and IL-18 are released from the dying cell, which can stimulate the production of ROS and recruit immune cells such as neutrophils that release MPO during inflammation. The decrease in MPO activity observed after KE consumption can, therefore, be explained by the inhibited release of IL-1β and IL-18, which in turn reduces the production of ROS and the recruitment of immune cells.

Furthermore, the administration of KE has been shown to significantly repress active caspase-3, confirming its antiapoptotic effects. Specifically, oxidative stress resulting from excessive ROS production in the damaged colon can overwhelm the cell’s antioxidant defenses, leading to DNA damage and mitochondrial dysfunction that trigger apoptosis. The release of cytochrome c from the mitochondria activates caspases and triggers the apoptotic pathway. Therefore, KE’s anti-apoptotic function may be attributed, at least in part, to its ability to improve the antioxidant defense machinery in the cell.

Our study shows that increased levels of β-hydroxybutyrate in rats with chronic colitis are positively correlated with tight junction proteins OCLN and CLDN5, with a strong tendency towards a positive correlation with ZO-1, indicating that increasing β-hydroxybutyrate levels could be beneficial in reducing gut permeability. Therefore, β-hydroxybutyrate may help manage colitis symptoms by stabilizing or upregulating these key barrier proteins, which may explain the beneficial effects of ketogenic diets or ketone supplementation in managing colitis and other forms of intestinal inflammation. Our analysis also revealed that β-hydroxybutyrate is negatively correlated with the measured proinflammatory cytokines in our study. Indicating that β-hydroxybutyrate may have anti-inflammatory effects in chronic colitis by reducing the production of proinflammatory cytokines and mitigating the inflammatory response. Furthermore, we found that β-hydroxybutyrate may help mitigate gut inflammation in chronic colitis by inhibiting NFκB and NLRP3 inflammasome activation and restraining oxidative stress as revealed also by correlation analysis. β-hydroxybutyrate may also enhance autophagy as revealed by reduced p62 and increased BECN1, which may help resolve gut inflammation in colitis. Finally, β-hydroxybutyrate may have a positive impact on maintaining a healthy microbiome.

In conclusion, this preclinical study indicates a potential therapeutic benefit of ketosis and ketone production in the management of various inflammatory diseases, particularly in the context of IBDs.

Our encouraging results provide a strong rationale for conducting further clinical research to explore the efficacy of β-hydroxybutyrate boosting compounds such as KEs in treating these conditions. If successful, these novel treatment approaches could significantly improve the lives of patients suffering from inflammatory diseases with the greatest safety. However, our study has limitations, including the use of an animal model that may not fully capture the intricate complexities of human pathophysiology, potentially limiting generalizability. The specific experimental conditions and the focus on short-term outcomes also restrict the applicability and understanding of the long-term effects of (R,R)-BD-AcAc2. Additionally, despite rigorous methodologies and blinded assessments, uncontrolled confounding variables should be considered. Therefore, more investment in clinical research is needed to fully realize the therapeutic potential of (R,R)-BD-AcAc2 and other KEs or ketone salts.

## 4. Materials and Methods

### 4.1. Drugs and Chemicals

(R)-3-Hydroxybutyl (R)-3-hydroxybutyrate ketone ester known as (R,R)-BD-AcAc2 with the formula C_8_H_16_O_4_ was obtained from MCE (Monmouth Junction, NJ, USA). Dextran sodium sulfate was supplied by Sigma-Aldrich (St. Louis, MO, USA; Mw = ~40,000). Fine chemicals and reagents were supplied by Sigma-Aldrich unless indicated else.

### 4.2. Animals

We obtained male Sprague Dawley (SD) rats from TBRI, aged seven weeks and weighing 180–200 g. The rats were given a two-week period to acclimate to the laboratory before the commencement of the experiment. They were kept in controlled environmental conditions since birth, with a temperature of 22 ± 2 °C, a relative humidity of 50 ± 10%, and a 12 h light/dark cycle (lights on from 09:00 a.m. to 09:00 p.m.). The rats were provided with ad libitum feeding and drinking. The protocol was approved by the IACUC at FPDU under FPDU24120,2. All animals were treated and sacrificed according to the corresponding guidelines.

### 4.3. Induction of Chronic Colitis in Rats

According to the procedure outlined by Hoffmann, et al. [32], chronic colitis was induced in the experimental animals by administering 2% DSS for seven days, followed by 1% DSS for ten days, and finally, another seven days of 2% DSS treatment. Throughout the experiment, the animals were monitored daily for changes in body weight, and the percentage of body weight loss was determined relative to their initial body weight on the first day of the study.

### 4.4. Experimental Design

Table 1 shows that the rats were randomly divided into six groups. The N group (n = 8) served as the control group. The N/F group (n = 8) consisted of rats that were subjected to 16 h of fasting including the dark cycle (from 05:00 p.m. to 09:00 a.m.) while being fed standard rodent food ad libitum at other times. The N/KE group (n = 8) consisted of rats that were given a mixture of KE and standard rodent food ad libitum. The CC group (n = 20) consisted of rats that received DSS. The CC/F group (n = 15) consisted of rats that received DSS and were subjected to the intermittent fasting protocol. The CC/KE group (n = 15) consisted of rats that received DSS and the KE/standard food mixture. On the start of day 25 at 09:00 a.m., all animals were sacrificed while under anesthesia (thiopental sodium 40 mg/kg).

In this experiment, blood samples were collected from all animal groups to determine β-hydroxybutyrate levels after 8 h of the fasting period, which begins at 5:00 p.m. on day 24. This occurred before the end of the day at 1:00 a.m. The animal groups were then allowed free access to food to prevent discrepancies in disease activity and body weight changes on the final day of the experiment. One hour after the end of the fasting period (at 10:00 a.m.), disease activity and body weight changes were assessed in all animal groups, followed by animal sacrifice and tissue sample collection. Throughout the experiment, body weight changes were monitored in all animal groups one hour after breakfast (at 10:00 a.m.) on previous days. It is worth noting that our preliminary trials revealed levels of β-hydroxybutyrate.

### 4.5. Rational of KE Dosing and Fasting Protocol

The normal control group of rats was given free access to standard rodent food (FPDU rodent food). In contrast, the groups receiving the KE were given (R,R)-BD-AcAc2 mixed with their standard rodent food ad libitum. Our pilot experiment showed that a 200 g SD rat in our lab typically consumes an amount of food equivalent to 7.5% of its body weight. Therefore, the rats in the KE group were given standard rodent food mixed with 4% (R,R)-BD-AcAc2 and 1% saccharin for palatability, as previously described [33], This mixture helped prevent food aversion in the rats. After conducting a preliminary trial, it was determined that the combination of KE and food resulted in increased plasma levels of β-hydroxybutyrate to >3 mmol/L after one week of continuous feeding. The amount of KE used in this experiment was then calculated accordingly. A plasma β-hydroxybutyrate level of >3 mmol/L indicates that the body is in a state of ketosis, this can occur in several situations, including during prolonged fasting and low-carbohydrate diets (such as the ketogenic diet). All animal groups received standard rodent food containing 1% saccharin. It is worth noting that our initial trials showed that the levels of β-hydroxybutyrate, measured at the end of the fasting period in both the fasting rat groups (N/F and CC/F), did not exceed 1 mmol/L. Moreover, there was no significant difference between these levels and those measured 8 h after the start of the fasting period. These findings indicate that a state of early ketosis was maintained until the end of the fasting period.

### 4.6. Assessment of the Weight/Length Colonic Ratio

To effectively evaluate the severity of colitis progression, one commonly used method is to calculate the weight/length colonic ratio. This approach involves measuring the entire colon that has been well-evacuated of feces and calculating the ratio of its weight to length. The resulting colon weight/length ratio is expressed in grams per centimeter of colonic tissue.

### 4.7. Assessment of the Disease Activity Score (DAI)

The Disease Activity Index (DAI) is an important tool used to evaluate the severity of colitis in preclinical studies. It provides a quantitative assessment of clinical signs associated with colitis, including alterations in body weight, consistency of stool, and blood in the stool. On the 25th day of the experiment, a blinded gastroenterologist determined the DAI. For each rat, the DAI was calculated as the average score for percentage body weight loss, diarrhea, and bloody stool. The evaluation criteria for body weight changes were: 0 for no weight loss, 1 for 1–5% loss, 2 for 6–10% loss, 3 for 11–15% loss, and 4 for 16–20% loss. For diarrhea, the evaluation criteria were: 0 for normal stool, 1 for soft stool, 2 for very soft stool, and 3 for watery diarrhea. The criteria for evaluating bloody stool were: 0 for negative hemoccult, 1 for positive hemoccult, 2 for traces of blood, and 3 for gross rectal bleeding.

### 4.8. Assessment of the Macroscopic Damage Index (MDI)

A blinded pathologist experienced in gastroenterology visualized the macroscopic features of colon tissue injury in the longitudinally opened colon segments. The MDI was determined for each rat using the following scoring criteria: a score of zero indicated no damage, a score of one indicated hyperemia without ulcers, a score of two indicated the presence of a linear ulcer without significant inflammation, a score of three indicated a linear ulcer with inflammation at one site, a score of four indicated the presence of two or more sites with inflammation or ulceration, a score of five indicated the presence of two or more major sites of inflammation or ulceration or one site with inflammation or ulceration extending ≥ 1 cm along the length of the colon, and a score of six-ten was assigned if the damage covered ≥ 2 cm along the length of the colon, with the score increasing by one for each additional centimeter of involvement.

### 4.9. Sample Collection and Preparation

After completing the experiment, the colons were weighed after being dissected and measured for length. Blood collected was used to isolate plasma for β-hydroxybutyrate analysis. The fresh colons were cleaned using ice-cold saline and dried using sterile towels. The colons were then partitioned into two parts. The first one, which included specimens from the distal colon, was kept in 4% neutral-buffered formalin to be histopathologically examined. The second part was instantly frozen in liquid nitrogen and stored at −80 °C for qRT-PCR, ELISA, and colorimetry. Additionally, 300 mg stool samples from the cecum were collected from each rat promptly after dissecting colons. DNA was extracted from the stool samples using the QIAamp DNA Stool Mini Kit (Qiagen Inc., Hilden, Germany) following the guidelines of the manufacturer. The extracted DNA was analyzed for concentration using a NanoDrop instrument (OPTIZEN NanoQ, Mecasys) via spectrophotometry.

### 4.10. Histological Examination

The colon tissues were washed with distilled water and then dehydrated using serial dilutions of alcohol. After that, the sections were washed in xylene and embedded in paraffin at 56 °C. Paraffin beeswax tissue blocks were then cut into 4–5-μm thicknesses using a microtome. The specimens were deparaffinized, rehydrated, and stained with hematoxylin and eosin stain. The standard histology procedures were performed blindly by a histologist, and the specimens were examined using a Leica DFC camera. The microscopic criteria of the histological scoring system of inflammation were applied as follows: 0 represented normal colonic tissue, 1 represented inflammation or focal ulceration limited to the mucosa, 2 represented focal or extensive ulceration and inflammation limited to the mucosa and submucosa, 3 represented focal or extensive ulceration and inflammation with involvement of the muscularis propria, 4 represented focal ulceration and transmural inflammation with involvement of the serosa, 5 represented extensive ulceration and transmural inflammation with involvement of the serosa, and 6 represented focal or extensive ulceration and transmural inflammation and perforation.

### 4.11. Determination of ROS, Malondialdehyde (MDA), Superoxide Dismutase (SOD), and Reduced Glutathione (GSH)

To investigate the presence of ROS in colon tissues, we followed a previously described methodology [13]. Initially, we homogenized 200 mg of fresh colon samples in ice-cold Tris-HCl buffer (40 mM, pH = 7.4) at a 1:10 *w*/*v* ratio. Afterward, we mixed 100 μL of homogenates with 1 mL of Tris-HCl buffer and added 10 μM of 2′,7′-dichlorofluorescin diacetate (Sigma). The reaction mixtures were incubated at 37 °C for 30 min, followed by measuring fluorescence intensity (FI) using a SpectraFluor Plus Microplate Reader (Tecan, Mainz, Germany) with excitation at 485 nm and emission at 525 nm.

To determine the concentration of MDA in colon tissue homogenate, a reaction was carried out with thiobarbituric acid (TBA) at 95 °C for 30 min under acidic conditions. This reaction produced a thiobarbituric acid reactive product, which was measured colorimetrically at 534 nm to determine the absorbance of the resulting pink product. For this procedure, a kit was utilized which was obtained from Bio-diagnostic (Giza, Egypt), and the analysis was conducted following the instructions provided by the manufacturer.

The SOD assay was performed by following the manufacturer’s instructions (Bio-diagnostic). This assay involved inhibiting the reduction of nitroblue tetrazolium dye mediated by phenazine methosulphate through the enzyme’s activity.

The method employed to determine GSH levels involved the reduction of 5,5′ dithiobis (2-nitrobenzoic acid) (DTNB) using GSH to yield a yellow-colored compound. The concentration of GSH was determined by measuring the absorbance of the reduced chromogen at 405 nm. The absorbance was directly proportional to the GSH concentration. All assays of oxidative stress markers were determined in duplicate.

### 4.12. Determination of Plasma β-Hydroxybutyrate

Plasma samples were centrifuged at 10,000× *g* for 10 min followed by centrifuging the supernatants at 10,000× *g* with a 50 KD ultrafiltration tube for 15 min. In this assay (Elabscience, Wuhan, China), β-hydroxybutyrate dehydrogenase catalyzes the oxidative dehydrogenation of β-hydroxybutyrate. Meanwhile, NAD^+^ is reduced to NADH which under the action of the hydrogen transmitter, transfers electrons to WST-8 to produce a yellow product. The content of β-hydroxybutyrate can be calculated by measuring the change of absorbance value at 450 nm. The β-hydroxybutyrate assay was determined in duplicate.

### 4.13. Determination of Tumor Necrosis Factor-Alpha (TNF-α), IL-6, IL-1β, IL-18, IL-10, and IL-4 Levels in Colon Tissue

The TNF-α and IL-10 levels were determined using ELISA kits purchased from LifeSpanBioSciences, Inc. (Seattle, WA, USA), while the IL-6 and IL-4 levels were assessed by kits from R&D System (Minneapolis, MN, USA). The IL-1β levels were measured by an ELISA kit obtained from BioLegend (San Diego, CA, USA), while the measurement of IL-18 was conducted using a kit obtained from eBioscience (Vienna, Austria). All protocols used in the assays strictly adhered to the manufacturer’s instructions. All cytokine assays were determined in duplicate.

### 4.14. Determination of Myeloperoxidase (MPO) Activity, NFκB DNA Binding Activity, Caspase-1 Activity, and Active Caspase-3

Myeloperoxidase is predominantly expressed in neutrophil granulocytes and is believed to mirror the level of neutrophil activation and infiltration into tissues. To determine the activity of MPO in the colonic tissue, we used an MPO assay obtained from Sigma-Aldrich. In this assay, hypochlorous acid produced by MPO reacts with taurine to form taurine chloramine, which subsequently reacts with TNB to produce DTNB. The activity of MPO was quantified as the amount of MPO enzyme required to hydrolyze the substrate and generate taurine chloramine that could consume 1.0 μmole of TNB per minute at 25 °C. We conducted the MPO assay in duplicate.

To evaluate the nuclear translocation of the p65 subunit, we utilized an assay kit obtained from Abcam that allowed for the analysis of nuclear extracts. The kit contained a specific double-stranded DNA sequence with the NFκB p65 consensus binding site (5′–GGGACTTTCC–3′) to selectively bind to active NFκB p65. We then used a primary antibody to detect an epitope of NFκB p65, which is only accessible when the protein is active and bound to its target DNA [34]. The NFκB p65 activity was determined in duplicate.

To assess the activity of caspase-1, we employed a caspase-1 colorimetric assay kit obtained from R&D Systems. This involved detecting the chromophore p-nitroanilide (p-NA) following its cleavage from the labeled substrate YVAD-p-NA, and quantifying the resulting p-NA light emission at 405 nm with a microtiter plate reader. Cytosolic extracts were obtained by preparing tissue lysates with chilled lysis buffer and then centrifuging them at 10,000× *g*. The protein concentration of the cytosolic extracts was determined, and 100 mg of protein was diluted in 50 μL of lysis buffer. Then, 50 µL of 2× reaction buffer (containing 10 mM DTT) and 5 μL of YVAD-p-NA substrate were added to each sample, and they were incubated at 37 °C for 1–2 h. Finally, we performed duplicate assays of the samples and read them at 405 nm in a microtiter plate reader.

We measured the levels of active caspase-3 using a kit obtained from MyBioSource Inc. (San Diego, CA, USA). The kit utilized a polyclonal anti-active caspase-3 antibody and an active caspase-3-HRP conjugate, and the color intensity obtained was inversely proportional to the amount of active caspase-3 present in the samples. The competition between the active caspase-3 from the samples and the active caspase-3-HRP conjugate for binding sites on the anti-active caspase-3 antibody was restricted by the limited number of available binding sites. As a result, as more binding sites were occupied by active caspase-3 from the sample, fewer binding sites remained available for active caspase-3-HRP conjugate to bind [35]. The determination of active caspase-3 was conducted in duplicate.

### 4.15. qRT-PCR Analysis for the mRNA Expression of Apoptosis-Associated Speck-like Protein Containing a CARD (ASC) and NLRP3

The extraction of total RNA from colonic tissues was performed using a Qiagen kit (Venlo, The Netherlands), following the supplier’s recommendations. RNA quality and purity were evaluated at 260 nm using a NanoDrop (Thermo Fisher Scientific, Waltham, MA, USA). To reverse transcribe RNA, the RevertAid First Strand cDNA synthesis kit was utilized. We conducted qRT-PCR using the StepOne™ Real-Time PCR System (Thermo Fisher Scientific). To determine relative expression, we calculated using the comparative cycle threshold (Ct) (2^−ΔΔCT^) method, normalized to the GAPDH gene. The PCR primer pairs used are described below: For ASC, F: 5′-CTCTGTATGGCAATGTGCTGAC-3′ and R: 5′-GAACAAGTTCTTGCAGGTCAG-3′. For NLRP3, F: 5′-GAGCTGGACCTCAGTGACAATGC-3′ and R: 5′-ACCAATGCGAGATCCTGACAACAC-3′. For GAPDH, F: 5′-TCAAGAAGGTGGTGAAGCAG-3′R: 5′-AGGTGGAAGAATGGGAGTTG-3′.

### 4.16. Determination of NLRP3 and NGSDMD

Kits supplied by MyBioSource Inc. (San Diego, CA, USA) were used for the determination of NLRP3 and NGSDMD in colon tissue homogenates according to the manufacturer’s instructions. The NGSDMD ELISA kit utilizes a competitive enzyme immunoassay technique. The assay involves incubating the assay sample and buffer with an NGSDMD-HRP conjugate and an anti-NGSDMD antibody in a pre-coated plate for one hour. Following incubation, the wells are decanted and washed five times before adding a substrate for the HRP enzyme. The enzyme-substrate reaction produces a blue-colored complex, which is stopped by the addition of a stop solution, resulting in a yellow solution. The intensity of the color is measured spectrophotometrically at 450nm in a microplate reader. The intensity of the color is inversely proportional to the concentration of NGSDMD, as both the NGSDMD from the sample and NGSDMD-HRP conjugate compete for binding sites on the anti-NGSDMD antibody. Since the binding sites are limited, the more NGSDMD from the sample that binds to the antibody, the fewer sites are available for NGSDMD-HRP conjugate to bind. By plotting a standard curve relating the intensity of the color to the concentration of standards, the NGSDMD concentration in each sample can be interpolated.

### 4.17. Determination of Beclin-1 (BECN1) and Sequestosome-1 (p62)

BECN1 and p62 colon tissue levels were determined using ELISA kits supplied by CUSABIO (Wuhan, China) and MyBioSource, respectively. All protocols followed the manufacturer’s instructions.

### 4.18. Determination of Zonula Occludens-1 (ZO-1), Occludin (OCLN), and Claudin-5 (CLDN5)

ZO-1, OCLN, and CLDN5 levels were determined following instructions given by CUSABIO.

### 4.19. Detection of Gut Microbiota Using Conventional PCR

To prepare the DNA samples for thermal cycling, we followed a protocol that involved creating reaction mixtures of 25 μL. The mixtures were prepared by combining 12.5 µL of my Taq red mix (Bioline Co., UK), 1 µL of each primer (10 µM each), 2.5 µL of DNA, and nuclease-free water. The PCR amplification protocol consisted of an initial denaturation step at 94 °C for 5 min, followed by 35 cycles at 94 °C for 30 s, annealing at a temperature that was calculated for each primer mix (as indicated in Table 2) for 30 s, extension at 72 °C for 45 s, and a final termination step at 72 °C for 3 min. To detect the expected PCR amplicons, we conducted electrophoretic separation on a 1.5% agarose gel and compared the results with a GeneRuler 100 bp plus DNA ladder (Thermo Scientific, USA). The gels were then stained with ethidium bromide and visualized using a UV transilluminator. For each type of bacteria, specific primer sequences are listed in Table 2.

### 4.20. qRT-PCR for the Detection of the Relative Abundance of Fusobacteria, Bifidobacteria, Bacteroides, Clostridium, and Lactobacillus

In order to evaluate the quantity of specific bacteria in the gut, we employed primers for the 16S rDNA housekeeping gene. Fecal DNA (40–80 ng) was extracted and combined with 12.5 μL (2 × SYBR Green PCR master mix (Willowfort Co., Birmingham, UK), 1.5 μL of each forward and reverse primer (10 μmol), and 7.5 μL of nuclease-free water, resulting in a final volume of 25 μL. Real-time PCR was performed on a MyGo machine using the following cycling protocol: an initial 5 min denaturation step at 95 °C, followed by 45 cycles of 95 °C for 20 s, annealing for 20 s, and 72 °C for 40 s. The Ct values and melting curves were obtained using MyGo software. The relative abundance of each bacterial species was calculated as a relative unit normalized to the total bacteria in the corresponding sample, using the 2^−ΔΔCt^ method (where ΔCt represents the average Ct value of each target minus the average Ct value of total bacteria). The primer sequences for detecting the various bacterial strains are listed in Table 2.

### 4.21. Statistical Analysis

The statistical analysis was carried out using the GraphPad Prism software version 9 (GraphPad Software Inc., La Jolla, CA, USA). The results were expressed as mean ± standard deviation (SD). One-way analysis of variance (ANOVA) was performed followed by Tukey’s post hoc test to determine the differences between groups. To investigate the correlation between multiple variables, Pearson correlation analysis was conducted. Survival probability was assessed by survival analysis comparisons, and survival curves were generated using the log-rank (Mantel–Cox) test. All statistical tests were performed at a significance level of less than 0.05.

## Figures and Tables

**Table 1:** Experimental design.

Exp. Groups	Days 1–7	Days 8–17	Days 18–24	Day 25
N group (n = 8)	Non-fasting	Non-fasting	Non-fasting	Sacrifice day
N/F (n = 8)	Fasting—16 h	Fasting—16 h	Fasting—16 h	
N/KE (n = 8)	Non-fasting KE	Non-fasting KE	Non-fasting KE	
CC (n = 20)	Non-fasting 2% DSS	Non-fasting 1% DSS	Non-fasting 2% DSS	
CC/F (n = 15)	Fasting—16 h 2% DSS	Fasting—16 h 1% DSS	Fasting—16 h 2% DSS	
CC/KE (n = 15)	Non-fasting 2% DSS KE	Non-fasting 1% DSS KE	Non-fasting 2% DSS KE	

CC, chronic colitis; DSS, dextran sodium sulfate; F, fasting; KE, ketone ester.

**Table 2:** Primer sequences for the detection of different species of bacteria.

Primer Name		Primer Sequence	Ta (°C)	bp
(16S)	F	GAGTTTGATCCTGGCTCAG	51	312
	R	GCTGCCTCCCGTAGGAGT		
*Fusobacterium*	F	GGATTTATTGGGCGTAAAGC	51.5	162
	R	GGCATTCCTACAAATATCTACGAA		
*Bacteroides* spp.	F	AAGGGAGCGTAGATGGATGTTTA	55	193
	R	CGAGCCTCAATGTCAGTTGC		
*Clostridium* spp.	F	CGGTACCTGACTAAGAAGC	50	429
	R	AGTTTGATTCTTGCGAACG		
*Bifidobacterium*	F	CTCCTGGAAACGGGTGG	51	551
	R	GGTGTTCTTCCCGATATCTACA		
*Lactobacillus* spp.	F	AGCAGTAGGGAATCTTCCA	50	334
	R	CACCGCTACACATGGAG		

## Data Availability

Data is contained within the article.

## Abbreviations

(R,R)-BD-AcAc2, (R)-3-Hydroxybutyl (R)-3-hydroxybutyrate ketone ester; ASC, apoptosis-associated speck-like protein containing a CARD; BECN1, Beclin-1; CLDN5, claudin-5; DAI, disease activity score; DSS, dextran sodium sulfate; GSDMD, gasdermin D; GSH, reduced glutathione; IBD, inflammatory bowel disease; KD, ketogenic diet; KE, ketone ester; MDA, malondialdehyde; MDI, macroscopic damage index; MPO, myeloperoxidase; NFκB, nuclear transcription factor kappa B; NGSDMD, gasdermin D N-terminal fragment; NLRP3, nucleotide-binding oligomerization domain-like receptor protein 3; OCLN, occludin; p62, sequestosome-1; ROS, reactive oxygen species; SOD, superoxide dismutase; TNF-α, tumor necrosis factor-alpha; ZO-1, zonula occludens-1.
