# Correction: Saber et al. BD-AcAc2 Mitigates Chronic Colitis in Rats: A Promising Multi-Pronged Approach Modulating Inflammasome Activity, Autophagy, and Pyroptosis. *Pharmaceuticals* 2023, *16*, 953

**DOI:** 10.3390/ph18010093

**Published:** 2025-01-14

**Authors:** Sameh Saber, Mohannad Mohammad S. Alamri, Jaber Alfaifi, Lobna A. Saleh, Sameh Abdel-Ghany, Adel Mohamed Aboregela, Alshaimaa A. Farrag, Abdulrahman H. Almaeen, Masoud I. E. Adam, AbdulElah Al Jarallah AlQahtani, Ali M. S. Eleragi, Mustafa Ahmed Abdel-Reheim, Heba A. Ramadan, Osama A. Mohammed

**Affiliations:** 1 Department of Pharmacology, Faculty of Pharmacy, Delta University for Science and Technology, Gamasa 11152, Egypt; sampharm81@gmail.com; 2 Department of Family Medicine, College of Medicine, University of Bisha, Bisha 61922, Saudi Arabia; malamri@ub.edu.sa; 3 Department of Child Health, College of Medicine, University of Bisha, Bisha 61922, Saudi Arabia; jalfaifi@ub.edu.sa; 4 Department of Clinical Pharmacology, Faculty of Medicine, Ain Shams University, Cairo 11566, Egypt; lobnasaleh_80@yahoo.ca; 5 Department of Clinical Pharmacology, Faculty of Medicine, Mansoura University, Mansoura 35516, Egypt; samghany@mans.edu.eg; 6 Human Anatomy and Embryology Department, Faculty of Medicine, Zagazig University, Zagazig 44519, Egypt; amaboregela@zu.edu.eg; 7 Basic Medical Sciences Department, College of Medicine, University of Bisha, Bisha 61922, Saudi Arabia; 8 Department of Histology and Cell Biology, Faculty of Medicine, Assiut University, Assiut 71515, Egypt; alshaima@aun.edu.eg; 9 Department of Anatomy, College of Medicine, University of Bisha, Bisha 61922, Saudi Arabia; 10 Department of Pathology, College of Medicine, Jouf University, Sakaka 72388, Saudi Arabia; ahalmaeen@ju.edu.sa; 11 Department of Medical Education and Internal Medicine, College of Medicine, University of Bisha, Bisha 61922, Saudi Arabia; mieadam@ub.edu.sa; 12 Department of Internal Medicine, Division of Dermatology, College of Medicine, University of Bisha, Bisha 61922, Saudi Arabia; aaljarallah@ub.edu.sa; 13 Department of Microbiology, College of Medicine, University of Bisha, Bisha 61922, Saudi Arabia; ameleragi@ub.edu.sa; 14 Department of Pharmaceutical Sciences, College of Pharmacy, Shaqra University, Shaqra 11961, Saudi Arabia; 15 Department of Pharmacology and Toxicology, Faculty of Pharmacy, Beni-Suef University, Beni Suef 62521, Egypt; 16 Department of Microbiology and Immunology, Faculty of Pharmacy, Delta University for Science and Technology, Gamasa 11152, Egypt; hebaaa.aadel@gmail.com; 17 Department of Clinical Pharmacology, College of Medicine, University of Bisha, Bisha 61922, Saudi Arabia

**Text Correction**

There was an error in the original publication [1]. Due to errors in the nomenclature of the tested drug by several suppliers, BD-AcAc2 was mistakenly referred to as (R,R)-BD-AcAc2, which is (R)-3-Hydroxybutyl (R)-3-hydroxybutyrate ketone ester with the formula C8H16O4. The correct nomenclature, however, is (R,S)-BD-AcAc2, which is R,S-1,3-Butanediol acetoacetate diester with the formula C12H18O6. Accordingly, BD-AcAc2 should be referred to as (R,S)-BD-AcAc2 throughout the manuscript, including in the title, keywords, and abbreviations.

A correction has been made to the Materials and Methods, Drugs and Chemicals, first sentence in paragraph 1:

BD-AcAc2, which is R,S-1,3-Butanediol acetoacetate diester with the formula C12H18O6, was obtained from MCE (Monmouth Junction, NJ, USA).

In the abbreviations section, “BD-AcAc2, (R)-3-Hydroxybutyl (R)-3-hydroxybutyrate ketone ester” was corrected as “BD-AcAc2, butanediol acetoacetate diester”.

The authors state that the scientific conclusions are unaffected. This correction was approved by the Academic Editor. The original publication has also been updated.
